# Early trauma deaths in a level 1 trauma center: whole-body 16-MDCT is associated with a threefold increase in the time interval from hospital access to emergency surgery if compared with a US-based protocol

**DOI:** 10.1186/cc9871

**Published:** 2011-03-11

**Authors:** G Nardi, E Cingolani, S Rogante, C Siddi, G Ranaldi, AP Cossu, D Piredda

**Affiliations:** 1 Ospedale S. Camillo-Forlanini, Rome, Italy

## Introduction

A retrospective analysis based on the data of the German Trauma Registry has shown a significant increase in the probability of survival in polytrauma patients submitted to whole body CT (WBCT). However, even when the CT is installed in the Emergency Department, the time required for positioning the patient for CT may significantly prolong the duration of CT diagnosis.

## Methods

Our hospital is a level 1 trauma center with a catchment population of 2.5 million; there are two CT scans in the ED. All severely injured patients are submitted to US on admission. WBCT is performed as the first-line radiologic investigation in haemodynamic-stable patients or in unstable patients with negative abdominal US and without a clear source of bleeding. Unstable patients with severe head trauma and lateral signs are also submitted to CT. To evaluate whether the use of CT in the severely unstable patients brings a significant delay in emergency surgery, we retrospectively analyzed all early trauma death from January 2009 to November 2010.

## Results

Seven hundred severe trauma patients (ISS >15) were brought in alive. Thirty-eight (5.4%) died before ICU admission: 21 died in the shock room before any surgical intervention. One patient was submitted to thoracotomy and laparothomy in the shock room and died. One more died on the CT table. The remaining 15 patients, severely hypotensive, were alive on admission to the OR. One of them was brought directly to the OR with no investigation because of massive bleeding from the extremities. The others had US on admission. US was the only investigation for seven of them; they all had a positive US showing important bleeding in the abdomen or in the chest. The other seven, who had no evidence of bleeding on the US, were submitted to WBCT. The mean time elapsed from hospital admission to OR entrance was 23 minutes (15 to 30 minutes) for patients who had only US and 70 minutes (52 to 90 minutes) for the CT group. The seven patients who had only US were all submitted to shock room decompressive minithoracotomy (five bilateral) with the suspicion of pneumothorax.

## Conclusions

Recent reports suggest implementation of multi-slice CT integrated into the resuscitation room, thus enabling resuscitation to be performed directly on the ER CT. Unless this new technology is adopted, even the ED-based CT still needs excessive time to be performed in most unstable patients. In a cohort of patients who eventually died in the OR, a diagnostic process including ED-based WBCT was associated with a threefold increase in the time needed from hospital admission to surgery.

